# Cenozoic megatooth sharks occupied extremely high trophic positions

**DOI:** 10.1126/sciadv.abl6529

**Published:** 2022-06-22

**Authors:** Emma R. Kast, Michael L. Griffiths, Sora L. Kim, Zixuan C. Rao, Kenshu Shimada, Martin A. Becker, Harry M. Maisch, Robert A. Eagle, Chelesia A. Clarke, Allison N. Neumann, Molly E. Karnes, Tina Lüdecke, Jennifer N. Leichliter, Alfredo Martínez-García, Alliya A. Akhtar, Xingchen T. Wang, Gerald H. Haug, Daniel M. Sigman

**Affiliations:** 1 Department of Geosciences, Princeton University, Princeton, NJ 08544, USA.; 2 Department of Earth Sciences, University of Cambridge, Cambridge, CB23EQ, UK.; 3 Department of Environmental Science, William Paterson University of New Jersey, Wayne, NJ 07470, USA.; 4 Department of Life and Environmental Sciences, University of California Merced, Merced, CA 95343, USA.; 5 Department of Environmental Science and Studies, DePaul University, Chicago, IL 60614, USA.; 6 Department of Biological Sciences, DePaul University, Chicago, IL 60614, USA.; 7 Sternberg Museum of Natural History, Fort Hays State University, Hays, KS 67601, USA.; 8 Department of Marine and Earth Sciences, Florida Gulf Coast University, Fort Myers, FL 33965, USA.; 9 Department of Atmospheric and Oceanic Sciences. Institute of the Environment and Sustainability, University of California, Los Angeles, CA 90095, USA.; 10 Emmy Noether Group for Hominin Meat Consumption, Max Planck Institute for Chemistry, 55128 Mainz, Germany.; 11 Senckenberg Biodiversity and Climate Research Centre, 60325 Frankfurt, Germany.; 12 Johannes Gutenberg University, Institute of Geosciences, 55128 Mainz, Germany.; 13 Department of Climate Geochemistry, Max Planck Institute for Chemistry, 55128 Mainz, Germany.; 14 Department of Earth and Environmental Sciences, Boston College, Chestnut Hill, MA 02467, USA.; 15 Department of Earth Sciences, ETH Zürich, CH-8092 Zürich, Switzerland.

## Abstract

Trophic position is a fundamental characteristic of animals, yet it is unknown in many extinct species. In this study, we ground-truth the ^15^N/^14^N ratio of enameloid-bound organic matter (δ^15^N_EB_) as a trophic level proxy by comparison to dentin collagen δ^15^N and apply this method to the fossil record to reconstruct the trophic level of the megatooth sharks (genus *Otodus*). These sharks evolved in the Cenozoic, culminating in *Otodus megalodon*, a shark with a maximum body size of more than 15 m, which went extinct 3.5 million years ago. Very high δ^15^N_EB_ values (22.9 ± 4.4‰) of *O. megalodon* from the Miocene and Pliocene show that it occupied a higher trophic level than is known for any marine species, extinct or extant. δ^15^N_EB_ also indicates a dietary shift in sharks of the megatooth lineage as they evolved toward the gigantic *O. megalodon*, with the highest trophic level apparently reached earlier than peak size.

## Teaser

Nitrogen isotope ratios in fossil teeth place extinct megatooth sharks at the top of the marine food web.

## INTRODUCTION

The ecology of ancient marine vertebrates is often investigated with fossil evidence of predator-prey interactions, such as bite marks, preserved stomach contents, or coprolites (*1*). More frequently, feeding strategies and diet are inferred from the morphological characteristics of fossils, such as jaw size or tooth shape [e.g., (*2*–*4*)]. Fossil evidence of predator-prey interactions can be rare and typically captures only a snapshot in time, while morphological characteristics tend to group taxa into broad categories and are related not only to current diet but also to the accumulated history of millions of years of evolution [e.g., (*5*)]. In addition, the co-occurrence of taxa informs ecological reconstruction but does not confirm interactions among taxa. These approaches provide initial hypotheses for ancient ecosystems and animals, but methodological advances provide new opportunities for geochemical diet proxies. In this study, we use a novel geochemical method to fill some of these gaps in our knowledge, with a focus on the diets of Cenozoic megatooth sharks.

Our understanding of ancient and modern animal ecology has increased with stable isotope analysis. The stable carbon, oxygen, and strontium isotope composition of fossil bones and tooth enamel is used to investigate primary producers in the food web; to distinguish terrestrial, aquatic, and marine habitats; and to reconstruct physiology [(*6*) and references therein]. The calcium isotope composition (δ^44/42^Ca) of tooth enamel(oid) and the zinc isotope composition (δ^66^Zn) of bone are emerging proxies for trophic level in the marine setting (*6*–*8*). However, the determination of trophic level from the fossil record is still poorly developed, particularly on million-year time scales.

The nitrogen isotope composition (δ^15^N) of animal tissues is a powerful and well-studied tool in identifying trophic level in modern ecosystems [e.g., (*6*)]. Animals require nitrogen from their diet. The δ^15^N of animal tissues in turn reflects the δ^15^N of their dietary nitrogen, but with a roughly 3‰ elevation, often referred to as the “trophic discrimination factor” (TDF) (*6*). The TDF arises from isotopic discrimination associated with nitrogen metabolism and excretion, where preferential excretion of ^14^N as waste leaves the tissue nitrogen elevated in δ^15^N (*6*). Because of this discrimination, the δ^15^N of an organism’s tissue can be used as an indicator of its diet and trophic position.

Despite this solid basis for application, δ^15^N has only been measured in relatively recent (<14,000 years) marine vertebrates, specifically on fossil bone collagen [e.g., (*9*, *10*)]. Collagen is not well preserved beyond a 10,000- to 100,000-year time scale because it is chemically labile and largely exposed, making it susceptible to alteration and loss during early diagenesis (*11*). Occurrences of million-year-old preserved collagen are rare [e.g., (*12*, *13*)]. As a result, the application of δ^15^N-based trophic level proxies has been limited to the recent past.

In this study, we build on the well-established practice of using δ^15^N to understand ecology and diet by introducing a new substrate for δ^15^N-based paleoecological reconstructions on million-year time scales: the nitrogen-containing organic matter bound within the enameloid mineral matrix of shark teeth. Enameloid is a highly mineralized bioapatite structure with <5 weight % organic matter, analogous to enamel although with some differences in formation, structure, and composition (*14*, *15*). Enameloid-bound organic matter is composed of residual proteins from tooth formation (*16*). The method relies on technical advances in the isotopic measurement of nanomole quantities of nitrogen (N), specifically the coupled oxidation-denitrifier method (*17*–*19*), which is necessary to measure the isotopic composition of the very low concentration of enamel(oid)-bound organic matter. Recent analyses have shown that, similar to other tissues, the δ^15^N of modern terrestrial mammal tooth enamel organic matter records diet and trophic level enrichment (*19*). In contrast to other fossil types, the apatite mineral of enamel(oid) is resistant to alteration (*11*, *20*). Furthermore, other mineral-bound organic matter proxies such as foraminifera carbonate have preserved δ^15^N signals over million-year time scales (*21*), suggesting that enameloid-bound organic matter might have a similar preservation potential, far beyond the time scales possible with collagen in bone and tooth dentin.

To ground-truth the δ^15^N of enameloid-bound organic matter (enameloid-bound δ^15^N, δ^15^N_EB_), we compare δ^15^N_EB_ and dentin collagen δ^15^N from modern sand tiger (*Carcharias taurus*) shark teeth. We then apply the δ^15^N_EB_ method to the fossil record to reconstruct the trophic ecology of a group of large megatooth sharks (genus *Otodus*) that evolved during the Cenozoic (~66 to 3.5 million years ago).

The most notable megatooth shark is *Otodus megalodon* of the family Otodontidae, with a conservative estimated maximum body length of 15 to 20 m (*22*, *23*), the largest known macrophagous shark. Its ancestors include other species of the genus *Otodus* that evolved from *Cretalamna* rooted in the Cretaceous (*24*). *O. megalodon* is well known in the fossil record from its large teeth, up to 16.5 cm total height (*23*), and it had a cosmopolitan distribution from the middle Miocene to the middle Pliocene (~16 to 3.5 million years ago) (*25*). Climatic and ecological causes [e.g., (*25*–*27*)] have been proposed for its extinction approximately 3.5 million years ago (*26*), but so far, there is limited evidence as to the ecology of megatooth sharks.

*O. megalodon* is widely assumed to have been an apex predator of the Neogene ocean. Its large, triangular, serrated teeth [e.g., (*2*)] and bite marks in fossil cetacean and pinniped bones suggest that adult *O. megalodon* had a diet of marine mammals [(*28*–*30*) and references therein]. While this evidence is compelling, the morphological trend observed in the megatooth shark lineage may not necessarily suggest any possible dietary preference or shift (*31*), and bite marks reflect brief events that may not represent the overall diet of *O. megalodon*. A high trophic level for *O. megalodon* has been inferred from low δ^44/42^Ca values of two Pliocene teeth (*7*); however, this evidence is so far limited in scope with respect to sample size, temporal span, and spatial distribution. Identifying the trophic position of these megatooth sharks is crucial for characterizing their ecology and testing hypotheses about their evolution and extinction that involve their reliance on or competition with specific marine mammal taxa [e.g., (*26*, *28*)].

## RESULTS AND DISCUSSION

### Analytical precision of δ^15^N_EB_ measurements

We assessed the analytical precision of the δ^15^N_EB_ measurement with a fossil enameloid standard that was run in triplicate alongside samples in every batch. For comparison, we also concurrently ran a coral carbonate standard that is regularly used as a general laboratory reference for mineral-bound δ^15^N measurements. The method for coral-carbonate–bound organic matter δ^15^N has been applied extensively, with analytical uncertainties that are well understood (*32*). For the same batches from 2017 to 2020, the long-term variability (1 SD) was 0.70‰ (average variability 0.37‰ within batches) for the fossil enameloid standard δ^15^N_EB_ and 0.29‰ (average variability 0.21‰ within batches) for the coral carbonate standard δ^15^N (fig. S1, A and B). In terms of nitrogen (N) content, the long-term variability was 0.62 μmol N/g (7.4%, average variability 0.38 μmol N/g within batches) for the fossil enameloid standard and 0.11 μmol N/g (5.4%, average variability 0.08 μmol N/g within batches) for the coral carbonate standard (fig. S1, C and D).

The higher δ^15^N_EB_ and N content variability of the fossil shark enameloid standard could be explained either by an intrinsic material property of enameloid that results in lower analytical precision or by the heterogeneous composition from preparation of the fossil enameloid standard. The long-term variability is higher than the variability within a batch of analyses, suggesting batch-to-batch effects of the enameloid cleaning: This is not captured by the nitrogen isotope standards used to correct our δ^15^N results. Despite these technical details, the analytical precision is sufficient, especially given the large magnitude of δ^15^N_EB_ differences we observe among modern and fossil enameloid specimens.

### Modern ground-truthing of the δ^15^N_EB_ proxy

**Fig. 1.**
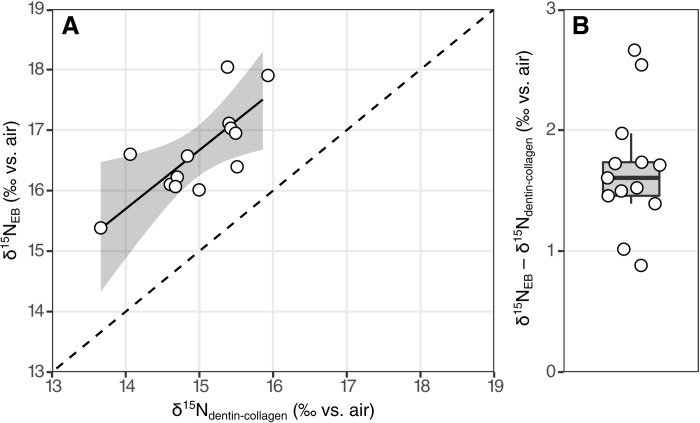
Comparison between δ^15^N_EB_ and dentin collagen δ^15^N. (**A**) δ^15^N_EB_ versus dentin collagen δ^15^N for 13 modern *C. taurus* teeth. Open circles show each measured sample, the solid line is a Deming regression with a bootstrapped 95% CI shown by the shaded gray region, and the dashed line is 1:1. (**B**) Difference between δ^15^N_EB_ and dentin collagen δ^15^N for the same measured samples (open circles) with a box plot of the distribution.

While dentin collagen is not a reliable source of organic nitrogen in ancient fossils, the δ^15^N of dentin collagen in modern sharks has been established as a trophic level proxy that robustly records variations in the dietary δ^15^N value (*33*, *34*). As dentin collagen and enameloid are formed over similar time frames, we expect that the δ^15^N values of these tissues should covary in response to diet and physiology. The δ^15^N_EB_ and dentin collagen δ^15^N values from 13 modern *C. taurus* teeth from different individuals are correlated (Pearson’s correlation 0.75, *t* = 3.76, df = 11, *P* = 0.0031; Fig. 1). The dentin collagen δ^15^N values range from 13.7 to 15.9‰, while δ^15^N_EB_ values range from 15.4 to 18.0‰. A Deming regression (total least squares regression accounting for the ratio of errors between δ^15^N_EB_ and dentin collagen δ^15^N) yields the relationship δ^15^N_EB_ ~ 0.97 [95% confidence interval (CI): 0.44 to 1.5] × δ^15^N_dentin-collagen_ + 2.1‰ (95% CI: −5.9 to 10.1‰) (Fig. 1A). The average offset between δ^15^N_EB_ and dentin collagen is 1.7 ± 0.5‰ (Fig. 1B).

The modern *C. taurus* δ^15^N values only span 2‰ but show promise in the close correspondence between enameloid-bound and dentin collagen δ^15^N (Fig. 1). The organic nitrogen within enameloid and dentin collagen is derived from diet and therefore might be expected to have closely overlapping δ^15^N values. The 1.7 ± 0.51‰ offset between the δ^15^N values of these tissues may be due to differences in amino acid composition. Shark enameloid mineralization occurs based on an organic protein matrix (*14*–*16*) and previous studies have identified noncollagenous protein within enameloid [(*15*, *16*) and references therein]. That protein is the main component of the enameloid-bound organic nitrogen and likely has a distinct amino acid composition from dentin collagen. This compositional difference may drive the δ^15^N of these tissues as amino acids can have distinct δ^15^N values [e.g., in sharks; (*35*)].

An additional consideration is the time over which enameloid-bound organic nitrogen and dentin collagen integrate the dietary δ^15^N signal. Enameloid mineralization precedes dentin formation and mineralization (*15*), with the exact timing sensitive to the rate of tooth replacement. The integration time will also be affected by the residence time of the tissue. A diet-switching experiment with captive leopard sharks (<1 m) had a residence time of 45 to 60 days for the carbon and nitrogen isotope composition of dentin collagen (*33*). Tissue incorporation rates scale metabolically (*36*, *37*); therefore, the megatooth sharks in this study would likely have longer residence times. The difference in integration time of enameloid-bound organic nitrogen and dentin collagen would tend to reduce the correlation between δ^15^N_EB_ and dentin collagen δ^15^N and may explain some of the variability of the observed offset of δ^15^N_EB_ from dentin collagen δ^15^N (Fig. 1B). However, it is unlikely to explain the observed δ^15^N offset between the two tissues.

Shark dentin collagen has a TDF of 2.0 to 2.8‰ (*33*), implying a TDF of approximately 4‰ for enameloid-bound organic matter. This enameloid TDF is consistent with a recent controlled feeding experiment that estimated TDF values for rodent tooth enamel bound organic matter δ^15^N between 1.9 and 4.9‰ (*19*). The δ^15^N_EB_ values of sharks can be related to δ^15^N measurements of other shark tissues (muscle, plasma, red blood cells, and fin) through their comparison to dentin collagen δ^15^N (*33*, *34*). Notably, dentin collagen δ^15^N is on average 1.9 ± 0.7‰ lower than muscle δ^15^N (*34*). Combined with our observations of δ^15^N_EB_ values that are on average 1.7 ± 0.5‰ higher than dentin collagen δ^15^N, this suggests that δ^15^N_EB_ should closely match shark muscle δ^15^N. Future studies should sample a broader range of dentin collagen δ^15^N and δ^15^N_EB_ to validate the offset we propose. In any case, the specimens in our study indicate a robust relationship between the δ^15^N values of these tissues and provide a framework to interpret trophic level from fossil shark teeth.

### Enameloid-bound δ^15^N through time

**Fig. 2.**
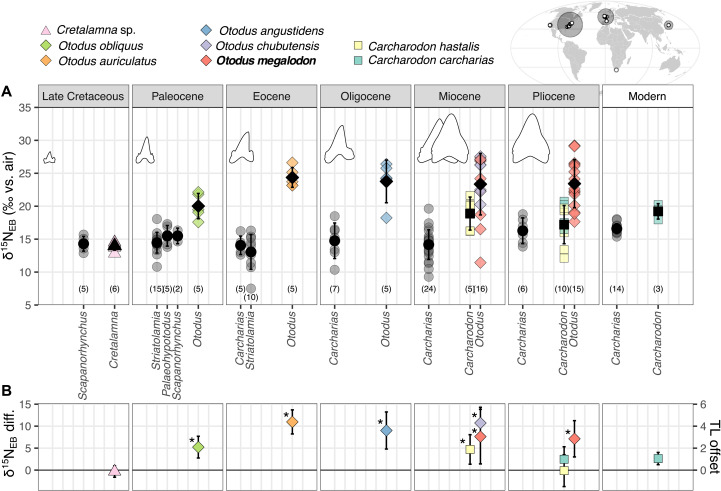
Shark enameloid-bound δ^15^N. (**A**) Shark δ^15^N_EB_ for each studied epoch, by taxon. Piscivorous shark teeth are plotted as gray circles. Otherwise, symbol shapes show the genus (triangle, *Cretalamna*; diamond, *Otodus*; square, *Carcharodon*) and colors show the species. Black symbols with error bars show the mean δ^15^N_EB_ ± 1 SD for each genus. Numbers indicate the number of teeth measured. Overlaid tooth diagrams are scaled to the estimated total length of each species (*22*). The map shows locations of sampled shark teeth; white symbols are collecting localities, and larger gray circles group the teeth into broad locations, with the size according to number of teeth. (**B**) Species-averaged δ^15^N_EB_ difference from contemporaneous piscivorous shark δ^15^N_EB_ ± 1 SD. The trophic level offset (right axis) is calculated from this δ^15^N_EB_ difference using a TDF of 2.5‰. Asterisks indicate species significantly different from contemporaneous piscivorous sharks (table S1).

We report δ^15^N_EB_ measurements for the modern *Carcharodon carcharias* and Neogene *C. carcharias*, *Carcharodon hastalis*, *Otodus chubutensis*, and *O. megalodon*, from a variety of localities (Fig. 2). In addition, we report δ^15^N_EB_ measurements for four *O. megalodon* ancestors: Late Cretaceous *Cretalamna* sp., Paleocene *Otodus obliquus*, Eocene *Otodus auriculatus*, and Oligocene *Otodus angustidens* (Fig. 2) (*24*). We compare these data to δ^15^N_EB_ measurements of taxa with piscivorous diets (*38*): Late Cretaceous *Scapanorhynchus* spp.; Paleocene *Striatolamia* spp., *Scapanorhynchus elegans*, and *Palaeohypotodus rutoti*; Eocene *Striatolamia macrota* and *Carcharias* sp.; Oligocene *Carcharias* sp.; Neogene *Carcharias* spp.; and modern *C. taurus* (Fig. 2). Distinct tooth morphologies and dentitions were used to infer prey preferences. In general, the teeth of piscivorous sharks are slender, elongated, and have smooth cutting edges, while those of macropredatory larger sharks, including megatooth sharks, are broad, robust, and serrated [(*3*) and references therein]. The average δ^15^N_EB_ of piscivorous sharks varies between 13.4 and 16.5‰ across all epochs. High δ^15^N_EB_ values are seen in Eocene *O. auriculatus* (24.4 ± 1.5‰), Oligocene *O. angustidens* (23.8 ± 3.2‰), Miocene *O. chubutensis* (24.9 ± 2.8‰), and *O. megalodon* from the Miocene (21.8 ± 5.8‰) and Pliocene (23.4 ± 3.6‰). Intermediate to these values are *O. obliquus* in the Paleocene (20.0 ± 1.9‰) and *C. carcharias* in the Pliocene (18.8 ± 2.1‰) and modern (19.2 ± 1.2‰). *C. hastalis*, which gave rise to extant *C. carcharias* (*39*), has similar δ^15^N_EB_ values to *C. carcharias* in the Miocene (18.9 ± 2.5‰) but lower, more piscivore-like values in the Pliocene (16.1 ± 3.5‰). The ancestor of the megatooth (*Otodus*) lineage, *Cretalamna* sp., has δ^15^N_EB_ values of 14.0 ± 0.7‰, similar to contemporaneous Late Cretaceous piscivorous sharks.

### Interpreting the fossil δ^15^N_EB_ signal

Before interpreting δ^15^N_EB_ values of fossil teeth in terms of trophic level, we address the geochemical integrity of δ^15^N_EB_ and the potential influence of baseline variations in δ^15^N. We explore the possibility for diagenetic alteration of the enameloid-bound organic matter by examining the N content of the enameloid. We found that the N content of fossil enameloid is on average lower than modern enameloid (4.8 ± 2.0 μmol N/g versus 7.4 ± 1.9 μmol N/g), although many fossil teeth have N content within the range of modern teeth (fig. S2, S3). *O. megalodon”,* have a higher N content relative to the other fossil enameloid samples, not a surprising finding given previous observations of species-specific differences in biomineral-bound N content [e.g., in foraminifera (*40*), fish otoliths (*41*), and tooth enamel (*19*)]. The lower N content of some fossil samples may suggest loss of organic matter from the enameloid matrix. If this loss occurred with substantial isotopic fractionation, we would expect a negative correlation between δ^15^N_EB_ and N content for these teeth as residual organic nitrogen would be left with a progressively higher δ^15^N value [e.g., in (*42*)]. However, this is not observed (fig. S2), and instead, we see a weak positive correlation in the fossil data overall with no significant correlation when considering the megatooth or piscivorous shark fossil data separately (fig. S2).

We hypothesize that the N content decline from modern to fossil enameloid occurred during early diagenetic maturation of the enameloid (e.g., small-scale recrystallization). During this process, a portion of the biomineral-bound organic matter could be exposed and fully degraded without preference for its chemical and/or isotopic composition and therefore have no substantial impact on δ^15^N_EB_, similar to what has been observed in modern foraminifera (*40*).

In addition to trophic position, the δ^15^N of animal tissues is influenced by the δ^15^N of nitrogen supplied to the base of the food web. This “baseline” δ^15^N signature is incorporated by autotrophs as they assimilate biologically available nitrogen from the environment, typically in the form of nitrate (NO_3_^−^) or ammonium (NH_4_^+^). Baseline δ^15^N can vary spatially (*43*) and through time [e.g., (*21*)] due to a variety of local and global nitrogen cycle processes in the ocean (*43*) and may complicate the interpretation of δ^15^N_EB_ as a trophic level proxy. Thus, considering baseline δ^15^N is important when reconstructing trophic level.

To constrain the potential impact of baseline δ^15^N variations on our δ^15^N_EB_ results, we examine the δ^15^N_EB_ values of the piscivorous sharks measured in this study. On the basis of their tooth morphology and comparisons to modern representatives, we assume that these piscivorous sharks maintained a similar diet and trophic level through time (*38*) and attribute δ^15^N_EB_ variations in these taxa to baseline δ^15^N variations propagating up the food chain. In the modern ocean, the δ^15^N values of large marine animals have been shown to directly reflect geographic patterns of baseline δ^15^N values (*44*), although an extensive temporal or geographic comparison has not been done for δ^15^N values in modern sharks. The δ^15^N measurements of individual amino acids can disentangle the trophic and baseline components of the δ^15^N signature in modern stable isotope ecology [(*35*) and references therein]. Unfortunately, this approach is not yet possible with enameloid-bound organic nitrogen due to analytical sample size requirements but could be an important path forward in the future upon methodological advancements. The use of piscivorous shark taxa, rather than taxa lower in the food web like foraminifera or bivalves, to constrain baseline δ^15^N is motivated by the notion that these sharks are more likely to share an environmental range and therefore integrate similar spatial and temporal variations of baseline δ^15^N as the *Otodus* sharks. Furthermore, there is added continuity in comparing δ^15^N values from the same substrate, enameloid-bound organic nitrogen.

From piscivorous taxa, we observe minimal change over time in the average δ^15^N_EB_ values (Fig. 2 and fig. S4). In addition, average δ^15^N_EB_ differences are <3‰ for genera that were sampled at multiple locations, and these differences are small relative to differences between piscivores and the other taxa, which are conserved across localities (fig. S5). From these piscivorous shark δ^15^N_EB_ data, we do not see evidence for large temporal or geographic baseline δ^15^N effects, and so, we interpret the δ^15^N_EB_ variations of the megatooth sharks in this study as dominantly a trophic level signal.

### Trophic level and diet of *Otodus megalodon*

*O. megalodon* δ^15^N_EB_ values are very high, with a large range of values in both the Miocene and Pliocene (Fig. 2A). Average δ^15^N_EB_ values for these two epochs are indistinguishable, and they are also consistent across locations; average *O. megalodon* δ^15^N_EB_ in Japan, North Carolina, and California are within 3‰ of each other, and the differences are not significant (fig. S5). *O. chubutensis*, which gave rise to *O. megalodon* (*2*), has similar δ^15^N_EB_ values (Fig. 2A).

There is a large range of δ^15^N_EB_ values for *O. megalodon* (Fig. 2A). We can generally rule out spatial δ^15^N variability as the cause for this large δ^15^N_EB_ range, as the differences between regions are small (fig. S5). The large δ^15^N_EB_ range may be driven by an ontogenetic shift, where larger *O. megalodon* occupy a higher trophic level, as seen in modern *C. carcharias* (*45*). However, with this dataset, it is unlikely that we can detect ontogenetic changes or that ontogenetic changes are driving the large spread in these δ^15^N_EB_ values of *O. megalodon*, as the samples studied here are mostly from mid-size individuals. We estimated the total animal length from the crown height of 12 fossil *O. megalodon* teeth that were also measured for δ^15^N_EB_. Estimated length ranged from 5.8 to 10.4 m, with an average length of 8 ± 1.5 m (fig. S6). We note that we often targeted fragmentary teeth in this study as we were performing destructive analyses, and so, our estimated sizes are less certain than those based on complete teeth. In any case, the estimated total lengths are all in the mid-size range of the overall size distribution of *O. megalodon* (*46*) and show no correlation to δ^15^N_EB_ (fig. S6).

There are two outstanding possibilities to explain the large range in *O. megalodon* δ^15^N_EB_. First, we may be overlooking high-frequency baseline δ^15^N changes that are not evident when analyzing the specimens grouped by epoch. This possibility could be investigated with more precise specimen age information. Alternatively, and our favored explanation, the large range of δ^15^N_EB_ values for *O. megalodon* may reflect a fundamental aspect of their ecology, specifically, a generalist diet, with individuals feeding across many prey types and different trophic levels. Modern ecological studies have shown large δ^15^N variations between individuals in many apex predators, including *C. carcharias* and other sharks, attributed to generalist feeding behavior [e.g., (*45*, *47*)]. As enameloid-bound organic matter integrates over relatively short time periods, we cannot distinguish between interindividual differences in diet preference and intraindividual generalist feeding behaviors.

The δ^15^N_EB_ of *O. megalodon* is, on average, 7.3‰ higher than that of contemporaneous piscivorous sharks (Fig. 2B) and also significantly higher than the largest extant macrophagous shark, *C. carcharias*, indicating prey with a particularly high δ^15^N. Estimated TDFs for shark muscle, the predominant tissue type in modern specimens, range from 2.3 to 5.5‰, with most values on the lower end of this range (*37*, *48*). Muscle TDFs are also relevant here because δ^15^N_EB_ values are approximately equivalent to muscle δ^15^N (see modern ground-truthing discussion above). On the basis of the full range of estimated TDFs (2.3 to 5.5‰), *O. megalodon* is, on average, 1.3 to 3.2 trophic levels above the piscivorous sharks, with a high likelihood that it was more than 2 trophic levels higher (Fig. 2B). Considering that the modern piscivore *C. taurus* has an estimated trophic level of 4.4 (*49*), this implies an average trophic level of 5.7 to 7.6 for the *O. megalodon* (Fig. 2B) and a trophic level range from 3.3 in the lowest δ^15^N_EB_ individual to 9.6 in the highest δ^15^N_EB_ individual, using a mid-range TDF of 2.5‰. This conclusion provides quantitative, integrative geochemical evidence of a very high, and flexible, trophic level for *O. megalodon*, and generally supports previous inferences from tooth morphology, fossilized bite marks on marine mammal bones, and tooth enameloid δ^44/42^Ca data (*2*, *7*, *28*–*30*).

**Fig. 3.**
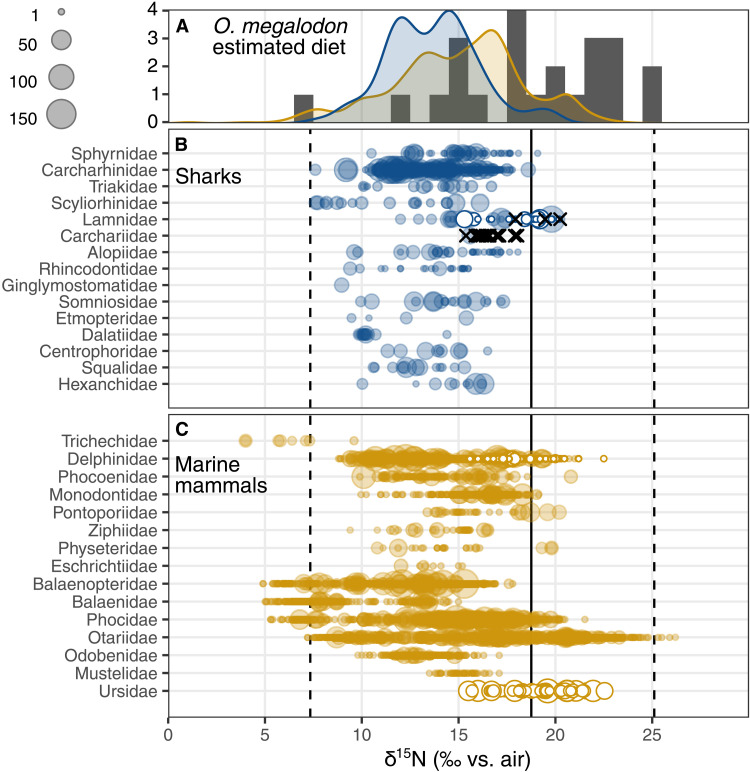
Estimated diet δ^15^N of *O. megalodon* compared to modern shark and marine mammal δ^15^N. (**A**) Gray bars show the estimated diet δ^15^N, calculated by subtracting the 1.7‰ offset between δ^15^N_EB_ and dentin collagen δ^15^N and a TDF of 2.5‰ from each *O. megalodon* δ^15^N_EB_ value. Curves show the distribution of modern shark (blue) and marine mammal (yellow) δ^15^N from the literature (data files S2 and S3). (**B**) Modern shark and (**C**) marine mammal δ^15^N plotted by family and sized by the number of individuals in each observation. Solid vertical line shows the average estimated diet δ^15^N of *O. megalodon*, and dashed vertical lines are the minimum and maximum estimated diet δ^15^N. Black x symbols in (B) are δ^15^N_EB_ measurements of modern *C. taurus* (family Carchariidae) and *C. carcharias* (family Lamnidae). In (B), *C. carcharias* are highlighted with white filled symbols, and in (C), polar bears (family Ursidae) and transient orcas (family Delphinidae) are highlighted with the same symbols.

To contextualize these findings, we estimated dietary δ^15^N from the *O. megalodon* δ^15^N_EB_ values by subtracting the 1.7‰ average offset between δ^15^N_EB_ and dentin collagen δ^15^N (Fig. 1B) and an average TDF of 2.5‰ associated with dentin collagen (*33*, *45*). This results in an estimated dietary δ^15^N of 18.8 ± 4.4‰ for *O. megalodon* (Fig. 3A). We compare this diet δ^15^N estimate to modern δ^15^N measurements of marine mammals and sharks from the literature (Fig. 3, B and C). The lower half of estimated dietary δ^15^N values corresponds well with the δ^15^N range of many marine mammal and shark species. On the other hand, the higher estimated dietary δ^15^N values are more difficult to match with the compilation of modern sharks and marine mammal δ^15^N values. Some marine mammal individuals do have δ^15^N values around the maximum *O. megalodon* estimated diet δ^15^N, predominantly eared seals (family Otariidae). The highest value, 26.2‰ for the bone collagen of a young South American sea lion (*Otaria flavescens*) (*50*), is higher than the highest estimated diet δ^15^N of *O. megalodon* by ~1‰ (Fig. 3). However, these high otariid δ^15^N values are driven in part by the locally highly elevated baseline δ^15^N off the east coast of South America (*50*). In addition, the reliance on a single regional species to explain the five highest *O. megalodon* δ^15^N_EB_ values is likely insufficient. The highest δ^15^N values outside of this region are from a group of polar bears (*Ursus maritimus*, family Ursidae) with an average δ^15^N of 22.5‰ (*51*), an unlikely prey item for *O. megalodon* given its habitat and origins in the Pleistocene (*52*), and an individual orca (*Orca orcinus*, family Delphinidae) from California with a δ^15^N value of 22.5‰ (*53*). However, these fail to reach the highest estimated dietary δ^15^N values of 25‰.

There are possible explanations that could reconcile the very high δ^15^N_EB_ *O. megalodon* with modern marine mammal δ^15^N values. First, Neogene marine mammals may have fed at higher trophic levels than do modern representatives, perhaps due to changes in diversity (*54*) or functional strategies (*55*). One example may be the presence of raptorial sperm whales in the Miocene (*4*). Second, *O. megalodon* may have had a unique feeding strategy, such as targeting nursing marine mammal pups or intraspecific cannibalism, both of which could have elevated its δ^15^N [e.g., (*56*, *57*)]. Cannibalism has been observed in modern sharks [e.g., (*58*)]. Third, we may have applied a TDF that is too small, artificially increasing estimated prey δ^15^N. While additional constraints will be necessary to establish exactly how individual *O. megalodon* achieved such elevated δ^15^N_EB_ values, *O. megalodon* was at a high trophic level that is not represented in modern ocean food webs. If we look to the highest trophic level marine mammal predators, transient orcas and polar bears, their δ^15^N values do not cover the range of estimated *O. megalodon* diet δ^15^N (Fig. 3), let alone the δ^15^N of the *O. megalodon* individuals themselves.

These results have implications for possible biotic extinction mechanisms of *O. megalodon*. For example, hypotheses for the extinction of *O. megalodon* have invoked changes in the diversity and size of baleen whales (mysticetes) (*26*, *28*). The high δ^15^N_EB_ values of *O. megalodon* indicate that baleen whales were not the dominant prey of *O. megalodon*, as modern baleen whales have a low trophic level and a correspondingly low δ^15^N (Fig. 3, families Balaenopteridae and Balaenidae). Given the fossil evidence for baleen, Miocene and Pliocene mysticete species likely had similar feeding patterns to their modern counterparts (*59*–*61*). These observations do not rule out second-order interactions between *O. megalodon* and baleen whales, but they do argue that the extinction of *O. megalodon* was not due to the loss of baleen whales as its main prey.

Another proposed biotic extinction mechanism involves competition, rather than prey availability, namely, that competition with the white shark (*C. carcharias*) drove the extinction of *O. megalodon* (*25*, *26*). The significant δ^15^N_EB_ differences between *O. megalodon* and *C. carcharias* indicate that large individuals of these species were likely not competing for the same diet (Figs. 2 and 3). However, it has been suggested that *C. carcharias* may have competed with juvenile *O. megalodon* for resources (*26*), which could be plausible given that we do not report δ^15^N_EB_ values for small (<5 m) *O. megalodon* in this study. We do note that the δ^15^N_EB_ values of Pliocene *C. carcharias* and Miocene *C. hastalis* are similar, which suggests that the onset of this possible competition dynamic is earlier than the extinction timing of *O. megalodon* 3.5 million years ago (Fig. 2) (*26*). A larger δ^15^N_EB_ dataset for *O. megalodon* and other shark taxa from the Miocene onward, especially with finer-scale age constraints and a focus on small individuals, may yield deeper insight into its demise in the Pliocene.

### Trophic evolution of the megatooth sharks

From the Late Cretaceous to the Neogene, the δ^15^N_EB_ of the megatooth (*Otodus*) lineage sharks diverges from that of contemporaneous piscivorous sharks (Fig. 2). Whereas *Cretalamna* sp. has similar δ^15^N_EB_ values to the piscivorous *Scapanorhynchus* spp. of the time, Paleocene *O. obliquus* δ^15^N_EB_ values are elevated by ~5‰ from contemporaneous piscivores, and in the Eocene, *O. auriculatus* values are elevated by 11‰ (Fig. 2B). This divergence in δ^15^N_EB_ reflects the evolution of megatooth sharks toward the very high trophic level of *O. megalodon* and supports previous interpretations of dietary shifts based on tooth morphology (*2*).

The initial increase in δ^15^N_EB_ values of the megatooth sharks occurs between the Cretaceous *Cretalamna* sp. and the Paleocene *O. obliquus*, before the emergence of marine mammals in the Eocene (*62*). This suggests that, at least for the megatooth sharks in the Paleocene, their evolution toward high trophic levels and larger size was disconnected from the evolutionary history of marine mammals. On the other hand, marine mammals were contemporaneous with the very high δ^15^N_EB_ Eocene *O. auriculatus* (*63*). This coincidence may indicate a connection between the evolutionary history of marine mammals and the transition from the Paleocene *O. obliquus* to the very high δ^15^N_EB_ values of Eocene through Pliocene *Otodus* species. Some Eocene cetacean taxa were likely feeding at a high trophic level, including eating smaller whales [e.g., (*64*, *65*)], which should have resulted in high tissue δ^15^N values. Predation on these taxa could have contributed to the very high δ^15^N_EB_ values of Eocene *O. auriculatus*. Further studies of fossil shark tooth δ^15^N_EB_ should help to disentangle the role of such macroevolutionary connections from oceanographic and climatic drivers of shark and marine ecosystem evolution over the Cenozoic.

**Fig. 4.**
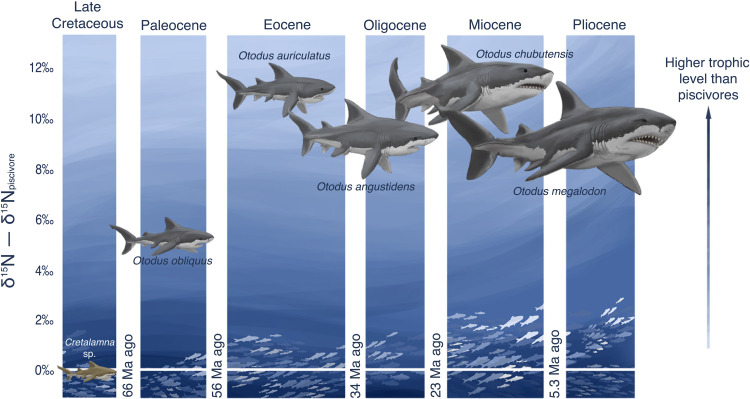
Trends in body size and trophic level of megatooth sharks (*Otodus*) through time, starting with their ancestor *Cretalamna* sp. While body forms of depicted sharks are hypothetical, they are sized relative to their estimated conservative maximum body size (*Cretalamna* sp. 3.5 m, *O. obliquus* 8 m, *O. auriculatus* 9.5 m, *O. angustidens* 11.5 m, *O. chubutensis* 13.5 m, *O. megalodon* 15 m) (*22*) and positioned vertically by their average δ^15^N_EB_ difference from contemporaneous piscivorous sharks. Ages are the boundaries between the geological time intervals (“Ma ago”, million years ago). Illustration by Christina Spence Morgan, copyright 2021.

Unexpectedly, the elevated δ^15^N_EB_ values of *O. megalodon* relative to contemporaneous piscivores had already been reached by *O. auriculatus* in the Eocene and were maintained in the Oligocene by *O. angustidens* (Fig. 2). These ancestors of *O. megalodon* had an estimated maximum length of at least 8 m but were markedly smaller than *O. megalodon* (Fig. 4) (*22*). This pattern suggests that the huge size of *O. megalodon* was not a necessary condition for its very high trophic level. Instead, it is possible that the very high trophic level contributed to allowing *Otodus* to evolve toward gigantism, which itself was encouraged by the benefits of regional endothermy and its embryos’ oophagy-based intrauterine cannibalism (*22*, *66*).

## MATERIALS AND METHODS

### Materials

This study examined 162 tooth specimens of modern and extinct sharks housed in the following three repository institutions (data file S1): Calvert Marine Museum (CMM), Solomons, MD, USA; San Diego Natural History Museum (SDNHM), San Diego, CA, USA; and United States National Museum of Natural History (USNM; Smithsonian Institution), Washington, DC, USA. The following individuals helped secure or loaned the reposited specimens for destructive sampling: M.A.B., G. Cliff, T. Deméré (SDNHM), S. Godfrey (CMM), D. Fox, K. Fujii, Y. Kurihara, H. Maisch, A. Millhouse (USNM), J. Nance (CMM), A. Sekita, K. Shimada, S. Tanaka, H.-D. Sues (USNM), D. Ward, B. Welton, and T. Yamamoto. For fossil specimens, primary literature sources describing the geology and paleontology of field localities for teeth examined in this study include the following: NC, Purdy *et al*. (*29*), Maisch *et al.* (*67*), Maisch *et al.* (*68*); Japan, Shimada and Shimada (*69*, *70*); and NJ, Maisch *et al.* (*63*). The exact stratigraphic horizon for USNM 431694-1, 431694-2, and 431694-3 (fossil *C. carcharias* teeth from Neuse River, NC, USA) is uncertain, but they are interpreted to have come from the Pliocene because *C. carcharias* is regarded as a post-Miocene taxon (*39*) and Pliocene marine deposits are common in the area (*71*).

### Enameloid-bound organic matter δ^15^N (δ^15^N_EB_)

The technique to measure δ^15^N_EB_ employs methods developed to analyze the δ^15^N of nanomole quantities of nitrogen (*17*, *72*) that have been applied to other modern and fossil biominerals such as foraminifera shells (*40*, *73*), diatom frustules (*18*), coral skeleton (*32*), fish otoliths (*74*), and mammalian tooth enamel (*19*) and now adapted for use with shark tooth enameloid.

Modern and fossil shark tooth enameloid powders were prepared by drilling from the enameloid layer, with care taken to avoid sampling the underlying dentin. Samples were drilled either at Princeton University (Princeton, NJ) or William Paterson University (Wayne, NJ).

Powdered enameloid samples were cleaned in three steps. Each sample was composed of approximately 20 mg of enameloid powders. Between each cleaning step, samples were rinsed three times with high-purity water (HPW). First, 10 ml of a 10% polyphosphate solution (adjusted to pH 8 with the stepwise addition of 4 N reagent grade HCl, ~2 ml) was added to samples in 15-ml centrifuge tubes, and samples were sonicated for 5 min to remove any clays or external detritus. Second, 10 ml of a sodium dithionite solution (31 g of sodium citrate + 10 g of sodium bicarbonate + 25 g of sodium dithionite in 500 ml of HPW, adjusted to pH 8 with the addition of 2 ml of 4 N reagent grade HCl) was added, and samples were placed in an 80°C water bath for 1 hour. The goal of this reductive cleaning step is to remove any oxidized coatings or contaminants. Third, samples were transferred to premuffled 12-ml borosilicate vials to which 5 ml of a basic potassium persulfate solution (2 g NaOH + 2 g potassium persulfate in 100 ml of HPW) was added, and samples were autoclaved at 120°C for 1 hour. This last cleaning step serves to oxidize and remove any organic matter external to the mineral structure of the enameloid. Samples were rinsed four times with HPW and dried down overnight in an oven at 60°C.

Between 2 and 5 mg of cleaned enameloid powders was weighed into premuffled 4-ml borosilicate vials. To dissolve the enameloid and release the enameloid-bound organic matter, 80 μl of 4 N HPLC grade HCl was added, and samples were allowed to sit for up to an hour until they appeared completely dissolved. To oxidize the organic matter to nitrate, 1 ml of a basic potassium persulfate solution (2 g of high-purity NaOH + 1 g of four times recrystallized potassium persulfate in 100 ml of low-temperature distilled HPW) was added, and samples were autoclaved at 120°C for 1 hour to promote oxidation. Two amino acid isotope references, USGS40 (δ^15^N = −4.5‰) and USGS65 (δ^15^N = 20.68‰) (*75*, *76*), and blanks were oxidized concurrently with the samples. Aliquots of these references with N amounts ranging from 5 to 50 nmol were added to premuffled 4-ml borosilicate vials and treated identically to the samples. These amino acid references allowed for isotopic blank corrections and served as a check that oxidation of the samples went to completion.

The high pH of the persulfate oxidation step precipitates the dissolved calcium. After oxidation, samples were centrifuged at 6000 rpm for 10 min, and the supernatant containing the sample nitrate was pipetted into a new precombusted 4-ml vial. Samples were then adjusted from basic conditions back to a pH of ~7 by stepwise additions of 4 N high-performance liquid chromatography (HPLC)–grade HCl.

Nitrate concentrations were measured by vanadium(III) reduction and chemiluminescence with a Teledyne NOx analyzer (*77*). The nitrate was then converted to nitrous oxide (N_2_O) by a strain of denitrifying bacteria (*Pseudomonas chlororaphis*) that lack N_2_O reductase activity; the “denitrifier method” is described in (*17*, *72*). The resulting N_2_O sample gas was analyzed with a custom-built automated N_2_O preparation system that extracts, purifies, concentrates, and delivers the N_2_O to a Thermo MAT253 stable isotope ratio mass spectrometer (*72*).

Isotope values of the analyzed sample nitrate are reported relative to air, calibrated by reference to two nitrate standards IAEA-NO3 (δ^15^N = 4.7‰) and USGS34 (δ^15^N = −1.8‰) (*78*, *79*). The δ^15^N of the blank associated with the oxidation procedure was calculated from the difference between the true δ^15^N and the measured δ^15^N values of the amino acid standards USGS40 and USGS65, and the sample δ^15^N is blank-corrected using this value and the measured size of the blank. Typical oxidation blank sizes are 0.3 to 0.5 nmol of nitrogen, representing <10‰ of the total nitrogen. Triplicates of in-house coral carbonate and fossil shark tooth enameloid standards are run with every batch to monitor the precision and accuracy of the coupled oxidation-denitrifier method. The long-term reproducibility of these in-house standards is 0.3 and 0.7‰ 1 SD, respectively. When possible, given the material constraints, replicate samples were run. As part of the isotope analysis, the N content for each sample in micromoles of nitrogen per gram of enameloid (μmol N/g) is also reported. All enameloid-bound δ^15^N and N content data from individual specimens as well as enameloid-bound δ^15^N and N content data from the fossil enameloid and coral carbonate standards are reported in data file S1.

### Dentin collagen δ^15^N measurements

Collagen was isolated from tooth dentin to determine the stable isotopic composition of organic nitrogen. Modern shark tooth powders were prepared by drilling from the dentin layer. First, 3 to 4 mg of sample powder was transferred to microcentrifuge tubes. Next, 1.5 ml of chilled 0.1 M HCl was added, and samples were allowed to demineralize under refrigeration for 25 min. After demineralization, samples were rinsed five times with deionized water and freeze-dried overnight. Samples were weighed out to 0.4 to 0.5 mg in 3 × 5 mm tin capsules. Collagen samples were measured for δ ^15^N values using a Costech 4010 Elemental Analyzer coupled to a Delta V Plus continuous flow isotope ratio mass spectrometer with a Conflo IV in the Stable Isotope Ecosystem Lab of (SIELO) University of California Merced. All data were corrected for linearity and drift using a suite of calibrated reference materials [USGS 40 (*n* = 9, δ^15^N = −4.52 ± 0.16‰); USGS 41a (*n* = 5, δ^15^N = 47.55 ± 0.08‰); Costech acetanilide (*n* = 4, δ^15^N = −0.38 ± 0.06‰)]. Dentin collagen δ^15^N values are reported in data file S1.

### *O. megalodon* size estimates

The total lengths of *O. megalodon* individuals were estimated from the tooth crown heights (CH) of the fossil teeth. We used Perez *et al.*’s (*2*, *23*) reconstructed dentitions of *O. chubutensis* and *O megalodon* based on a disarticulated but associated tooth set to determine the approximate tooth position of each of our *O. megalodon* samples and to determine the missing portion in the case of fragmentary specimens. This process led to the determination of the approximate tooth position for a total of 12 tooth specimens.

For total length estimations, we used Shimada’s (*80*, *81*) linear functions that represent CH–to–total length relationships of extant *C. carcharias*. Perez *et al.*’s (*23*) total length estimation method, which also requires additional assumptions and knowledge of the crown width or its ratio with the crown height, was not readily applicable to our samples because many of them were fragmentary, making their crown width too uncertain. Among the 12 tooth specimens, CMM-V-10506, 10971, and 10974 were identified as upper anterior teeth because of their tall symmetrical, broad (= nonconstricted) crown, and Shimada’s (2019) linear function for upper anterior teeth (“U”), found to still give robust total length estimates (*23*), was used (*81*). CMM-V-10505 was identified as a lower anterior tooth because of its tall symmetrical but constricted crown, and Shimada’s (*81*) linear function for lower anterior teeth (“L”) was used. CMM-V-10507, 10508, 10509, 105968, 10970, 10973, and SDNHM 143306-C were identified to be equivalent to one of mesially located upper lateral teeth (specifically the third through sixth teeth in the reconstructed dentition of Perez *et al.*) (*2*, *23*) because of their asymmetrical (i.e., inclined) but broad crown showing a height similar to its width. Because the exact tooth position of these seven specimens could not be determined decisively, we used Shimada’s (*80*) linear function for the upper second lateral tooth (“L2”) that yielded the most conservative total length estimations among the mesially located upper lateral teeth. CMM-V-10502 with a symmetrical but constricted crown was determined to be equivalent to the second or third lower lateral tooth in *C. carcharias*, and we used Shimada’s (*80*) function for the second lower lateral tooth (“l2”), which gave a more conservative total length estimate relative to that based on the third lower lateral tooth. The estimated total length for each of these 12 specimens is reported in data file S1.

### Shark and marine mammal literature δ^15^N values

Modern shark tissue δ^15^N measurements were compiled from the literature. Web of Science topic searches for [nitrogen isotope shark] were retrieved (156 results as of 24 March 2021). Of these, 106 had relevant modern shark δ^15^N data: Data from samples older than ~1900, methods-focused studies with only one or two individuals, compound-specific analyses, captive animals, and data reported in previous studies were considered not relevant. The database was then compiled from 64 papers for which the δ^15^N data were available either in supplement tables or in in-text tables, or summarized with a mean and SD in the text.

Modern marine mammal tissue δ^15^N measurements were similarly compiled from the literature. Web of Science topic searches for [marine mammal nitrogen isotope] (293 results as of 24 March 2021), [nitrogen isotope and (whale OR dolphin OR porpoise OR sea lion OR seal OR walrus OR otter) NOT (marine mammal)] (488 results as of 24 March 2021), and [nitrogen isotope AND (polar bear OR sirenian OR manatee OR dugong)] (84 results as of 1 October 2021) were retrieved. The second searches were done to access papers that focus on a specific family of marine mammals and do not include the term “marine mammal,” meaning that they were missed in the original search. Of these results, 330 had relevant δ^15^N data; data from samples older than ~1900, methods-focused studies, compound specific analyses, river dolphins, captive animals, and data reported in previous studies were all excluded. The database was then compiled from 225 papers for which the δ^15^N data were available either in supplement tables or in in-text tables, or summarized with a mean and SD in the text.

For both the shark and the marine mammal literature compilations, the most individualized information possible was recorded: If a paper reported the isotope values by individual shark or marine mammal, either in the main text or in the supplementary materials, these were added to the database as unique observations. In many cases, papers only included summarized statistics for a group of individuals, in which case this information, along with the number of individuals and the standard deviation, was included as an observation. In addition, a variety of collection, tissue type, and taxonomic information were recorded. These two databases, descriptions of variables, and lists of citations are provided in data files S2 and S3.

### Data analysis

Data analysis and visualization were done with R statistical software (*82*) using the tidyverse (*83*) and BSDA (*84*) packages. We report averages ±1 SD unless otherwise stated. The regression between δ^15^N_EB_ and dentin collagen δ^15^N was done with a bootstrapped Deming regression, with the ratio of errors being set at 3.5 (0.7‰ 1 SD for δ^15^N_EB_ measurements and 0.2‰ 1 SD for dentin collagen δ^15^N measurements). The δ^15^N_EB_ difference in Fig. 2B was calculated by subtracting the average of all piscivore shark δ^15^N_EB_ from every other shark species average δ^15^N_EB_, for each epoch. Errors were fully propagated. Welch’s *t* tests were used to compare species δ^15^N_EB_ with the δ^15^N_EB_ of contemporaneous piscivore sharks (Fig. 2B and table S1).
