# Supplementary material for: Cenozoic megatooth sharks occupied extremely high trophic positions
Source: Sci Adv. 2022 Jun 22;8(25):eabl6529. doi: 10.1126/sciadv.abl6529 (PMC9217088; doi:10.1126/sciadv.abl6529)
Supplement: Supplementary file 1 — Figs. S1 to S6 Table S1 [file sciadv.abl6529_sm.pdf]

Science Advances
AAAS

Supplementary Materials for

Cenozoic megatooth sharks occupied extremely high trophic positions

Emma R. Kast *et al.*

Corresponding author: Emma R. Kast, emma.r.kast@gmail.com

Sci. Adv. **8**, eabl6529 (2022)
DOI: 10.1126/sciadv.abl6529

The PDF file includes:

Figs. S1 to S6
Table S1
Legends for data S1 to S3

Other Supplementary Material for this manuscript includes the following:

Data S1 to S3

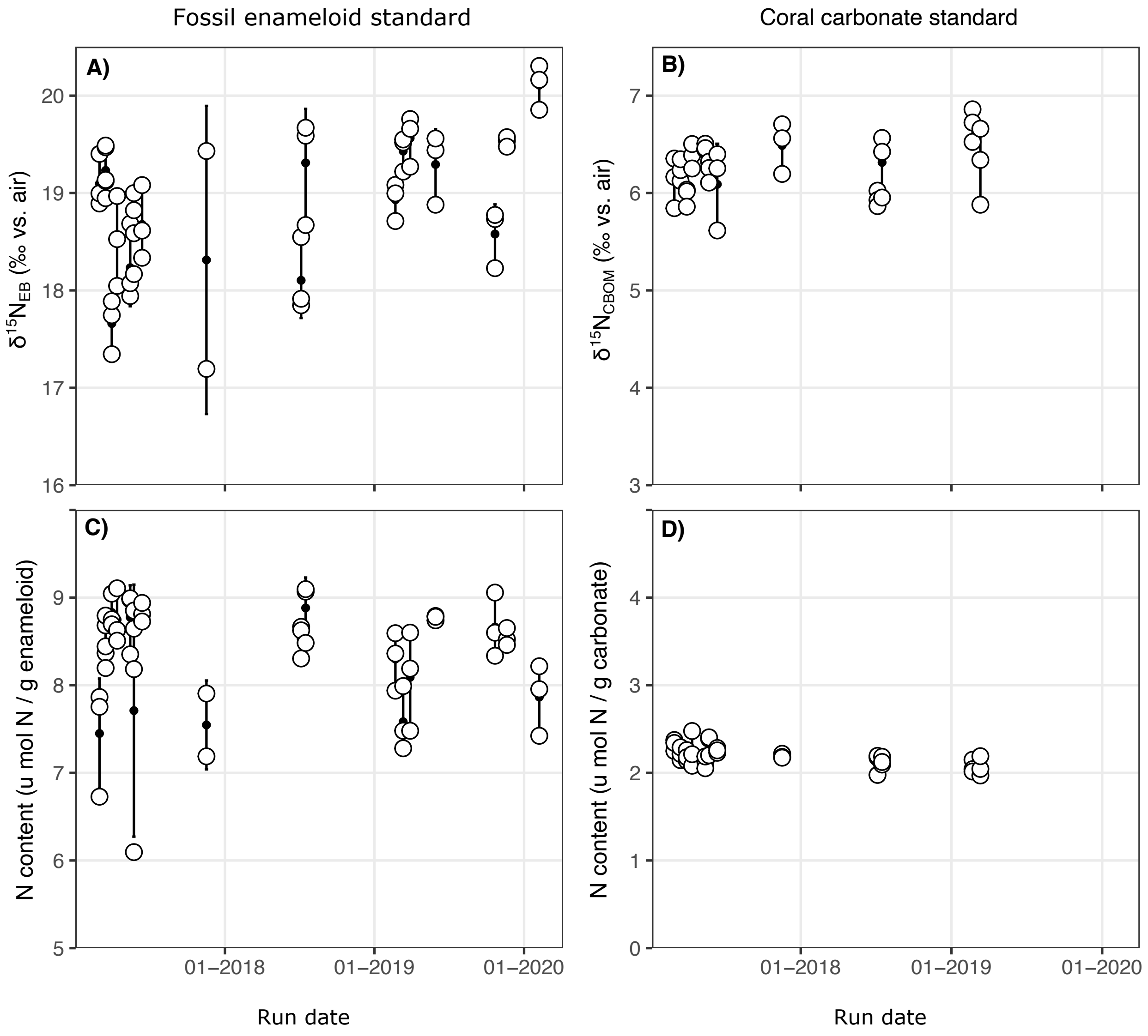

Fig. S1.
Results from fossil enameloid and coral carbonate standards run in triplicate with each batch of analyses. (**A**) $\delta^{15}N_{EB}$ and (**C**) N content for the fossil enameloid standard by analysis date; (**B**) carbonate bound organic matter $\delta^{15}N$ ($\delta^{15}N_{CBOM}$) and (**D**) N content for the coral carbonate standard. Note that the scales for $\delta^{15}N_{EB}$ and $\delta^{15}N_{CBOM}$, and N content have different values but the same range to allow for comparisons between the two standards. The coral carbonate standard was exhausted in mid 2019 and replaced with a different coral carbonate standard and so does not extend over the same range of analysis dates.

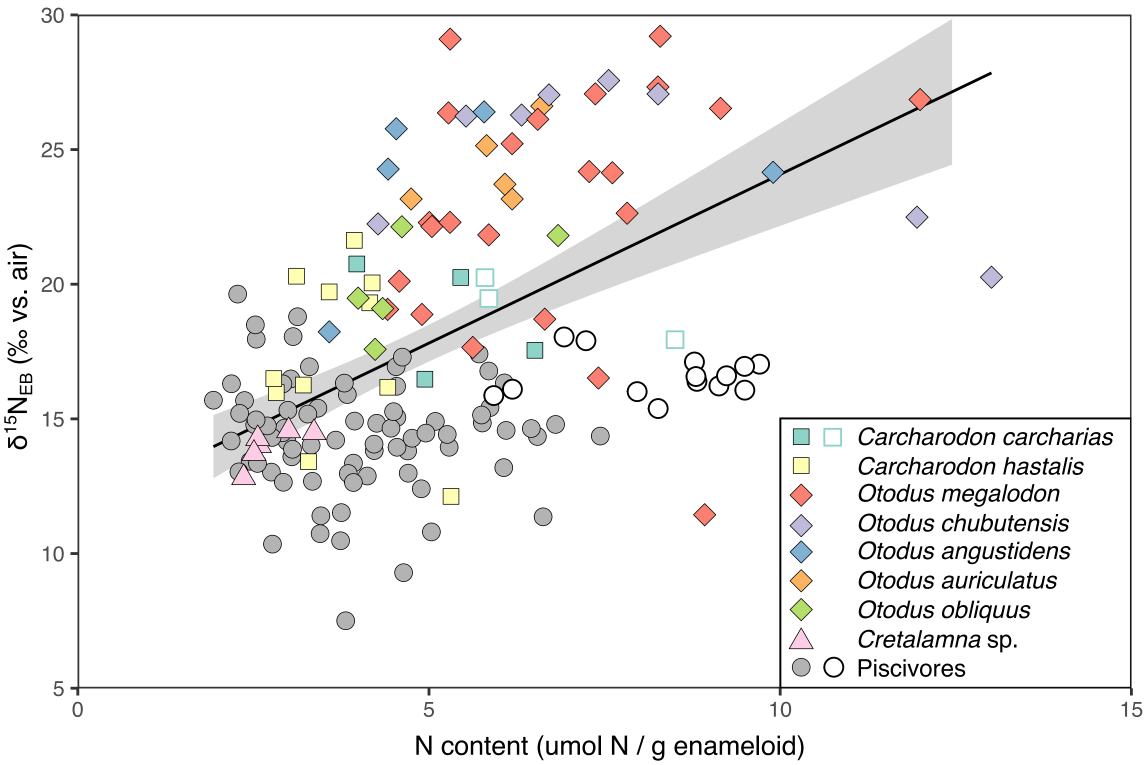

Fig. S2.
$\delta^{15}N_{EB}$ vs. N content for all specimens measured in this study. Symbols in legend and as in Fig. 2A, with the exception that modern specimens (*Carcharodon carcharias* and *Carcharias taurus*) are shown here in open symbols for reference. As described in the text, we would expect a negative correlation between $\delta^{15}N_{EB}$ and N content if diagenesis is altering the $\delta^{15}N_{EB}$ values of these specimens. There is a weak positive correlation across all fossil specimens (black line and shaded 95% CI, $r^2 = 0.27$, Pearson's correlation 0.52, t = 7.23, df = 144, p-value = $2.1*10^{-11}$). However, this is driven mostly by the high $\delta^{15}N_{EB}$ and high N content of *Otodus* sharks relative to the other fossil specimens, suggesting that there may be taxa specific differences in N content of shark enameloid. When considering *Otodus* teeth or piscivore teeth alone, there is no significant correlation between $\delta^{15}N_{EB}$ and N content (Pearson's correlation 0.17, t = 1.16, df = 44, p-value = 0.25 and Pearson's correlation -0.09, t = -0.82, df = 77, p-value = 0.42, respectively). Overall we do not find evidence from the paired $\delta^{15}N_{EB}$ and N content data for a diagenetic influence on the $\delta^{15}N_{EB}$ values of these specimens.

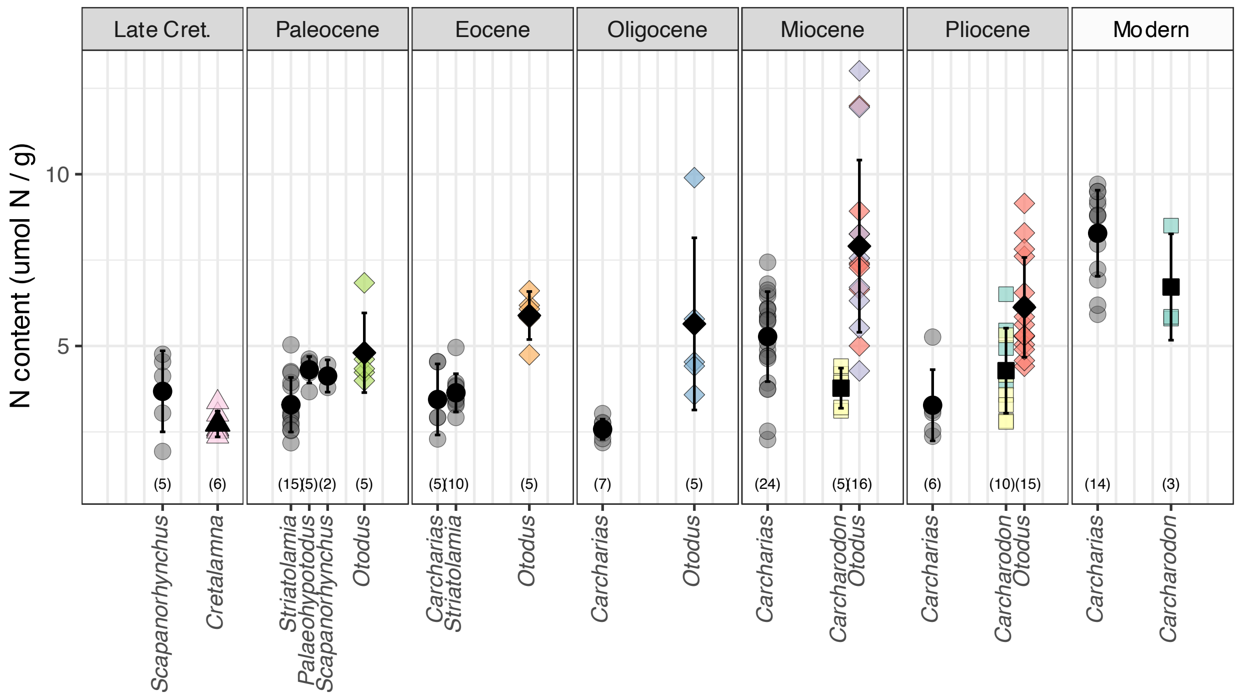

Fig. S3.

Shark enameloid-bound N content for each studied epoch, by taxa. Symbols as in Fig. 2A. Lower N content in fossil samples may be due to the loss of organic matter from the enameloid matrix over time. Alternatively, there may be a role for contamination by tooth dentin which tends to lose its organic matter quickly. This would tend to bias the fossil samples to lower N content without significant changes in $\delta^{15}N_{EB}$. With the exception of higher N content in *Otodus megalodon* tooth enameloid, there does not seem to be any consistent patterns in enameloid-bound N content of fossil teeth over the Cenozoic epochs studied here.

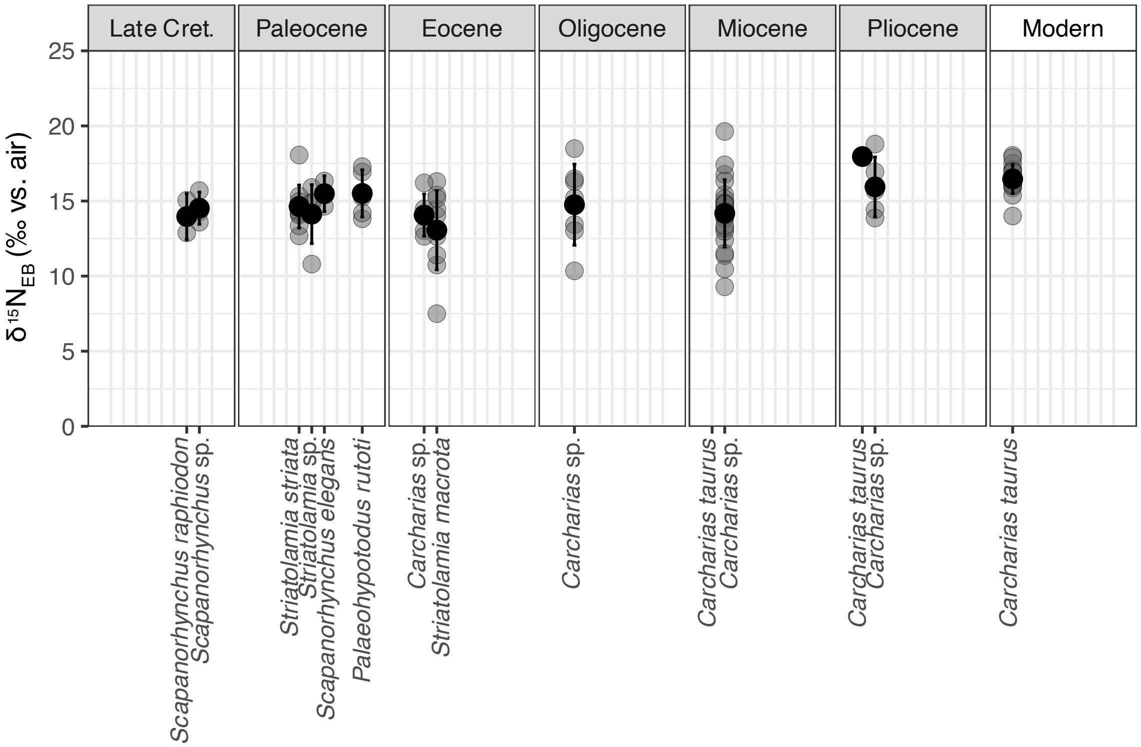

Fig. S4.

Assessing baseline $\delta^{15}N$ changes. Piscivorous shark $\delta^{15}N_{EB}$ for each studied epoch, by species. Mean $\delta^{15}N_{EB} \pm 1\sigma$ are given in black symbols for each species.

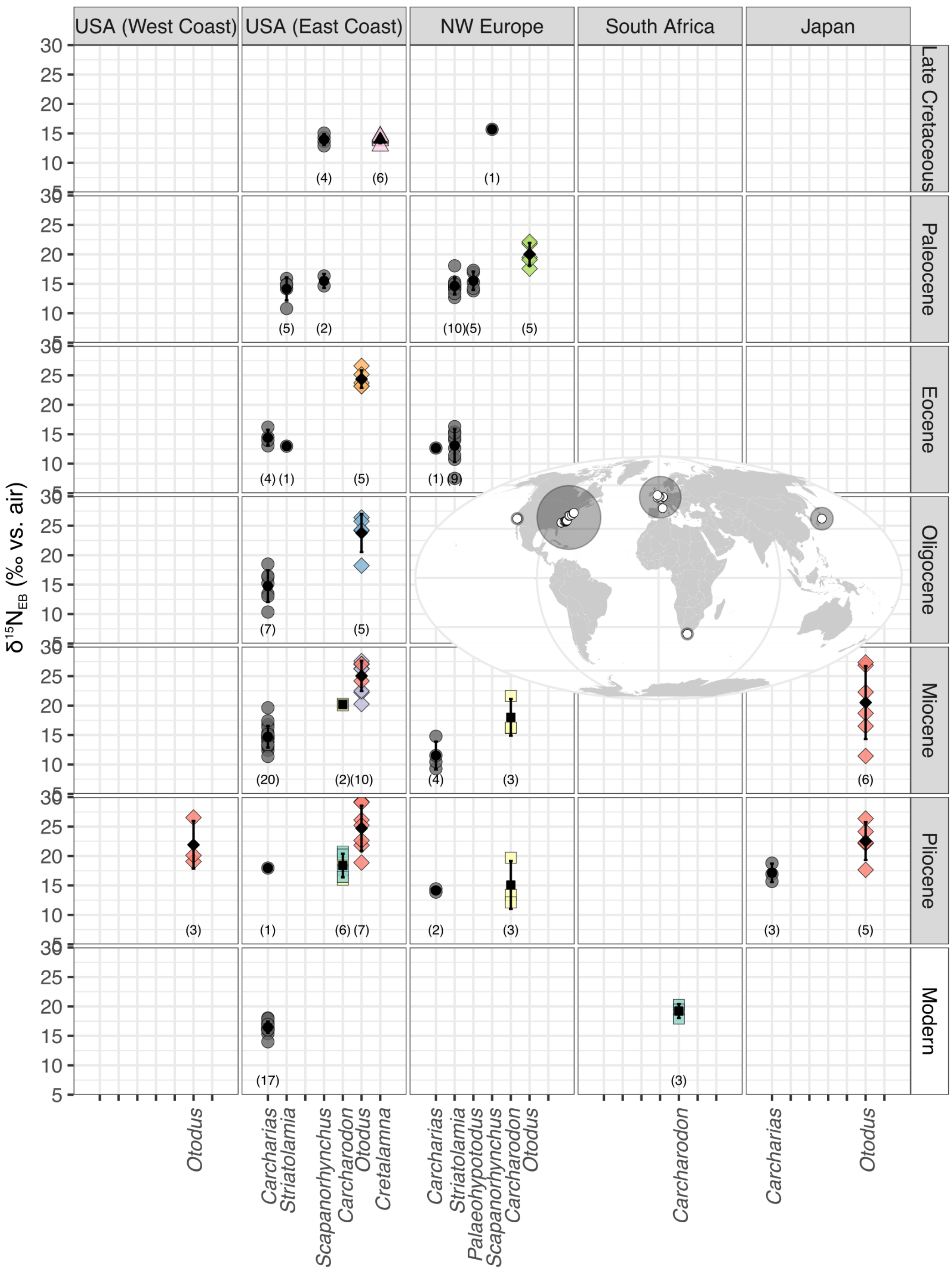

Fig. S5.
Shark $\delta^{15}N_{EB}$ for each studied epoch and broad location, by taxa. Symbols and inset map as in Fig. 2. Differences between average genera $\delta^{15}N_{EB}$ at different locations is < 3‰, and patterns between genera are conserved across locations.

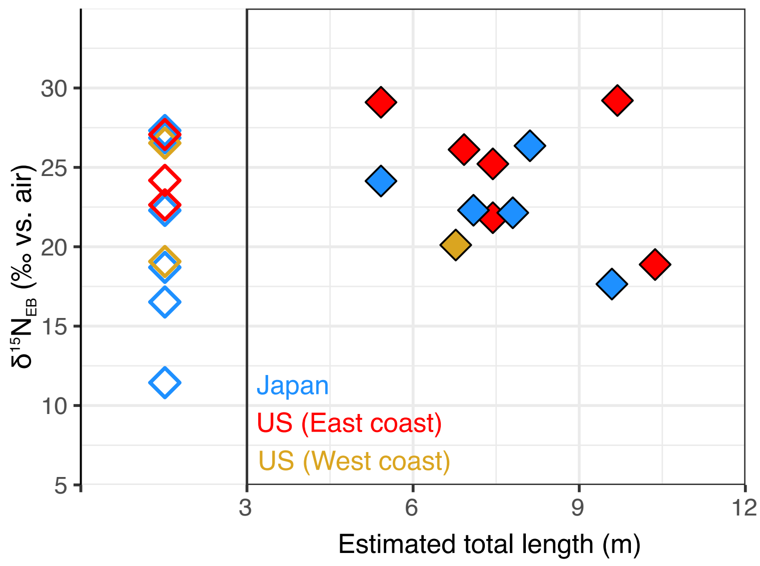

Fig. S6.

Otodus megalodon $\delta^{15}N_{EB}$ vs. total length as estimated from tooth size. Colors are location: USA east coast (red, n = 6), Japan (blue, n = 5), and USA west coast (yellow, n = 1) (see Data S1). *O. megalodon* $\delta^{15}N_{EB}$ samples without size estimates are with empty symbols to the left. *O. megalodon* was likely ~ 2 m at birth (46).

Table S1.

Results of Welch's t-test comparisons of the $\delta^{15}N_{EB}$ of large shark species and contemporaneous piscivorous sharks, for each epoch. Late Cretaceous *Cretalamna* sp., Pliocene *Carcharodon carcharias*, Pliocene *C. hastalis*, and modern *C. carcharias* are indistinguishable from contemporaneous piscivorous sharks.

epoch	species	t	df	p
Late Cretaceous	*Cretalamna* sp.	-0.57	6.16	0.5886
Paleocene	*Otodus obliquus*	5.68	5.27	0.0020
Eocene	*Otodus auriculatus*	12.26	10.84	0.0000
Oligocene	*Otodus angustidens*	5.09	7.68	0.0011
Miocene	*Carcharodon hastalis*	3.88	5.41	0.0100
Miocene	*Otodus chubutensis*	9.86	10.19	0.0000
Miocene	*Otodus megalodon*	3.63	7.71	0.0071
Pliocene	*Carcharodon carcharias*	1.89	6.29	0.1054
Pliocene	*Carcharodon hastalis*	-0.07	8.52	0.9448
Pliocene	*Otodus megalodon*	5.79	16.85	0.0000
Modern	*Carcharodon carcharias*	3.72	2.37	0.0501

Data S1. (separate file)

Specimen information, enameloid-bound $\delta^{15}N$, and N content data for modern and fossil teeth used in this study. Tooth sizes and estimated total lengths for *Otodus megalodon* specimens. Dental collagen $\delta^{15}N$ values for modern *Carcharias taurus* specimens. Enameloid-bound $\delta^{15}N$ and N content for fossil enameloid standard analyses, carbonate-bound $\delta^{15}N$ and N content data for coral carbonate standard analyses.

Data S2. (separate file)

Literature compilation of modern shark $\delta^{15}N$ measurements, including description of variables and list of citations.

Data S3. (separate file)

Literature compilation of modern marine mammal $\delta^{15}N$ measurements, including description of variables and list of citations.
